# Gestational diabetes mellitus in women increased the risk of neonatal infection via inflammation and autophagy in the placenta

**DOI:** 10.1097/MD.0000000000022152

**Published:** 2020-10-02

**Authors:** Yi-xiao Li, Deng-lu Long, Jia Liu, Di Qiu, Jingyun Wang, Xin Cheng, Xuesong Yang, Rui-man Li, Guang Wang

**Affiliations:** a The First Affiliate Hospital of Jinan University; b International Joint Laboratory for Embryonic Development & Prenatal Medicine, Division of Histology and Embryology, Medical College; c Key Laboratory for Regenerative Medicine of the Ministry of Education, Jinan University, Guangzhou, China.

**Keywords:** gestational diabetes mellitus, placenta, inflammation, autophagy, NF-κB

## Abstract

**Background:**

Gestational diabetes mellitus (GDM) produces numerous problems for maternal and fetal outcomes. However, the precise molecular mechanisms of GDM are not clear.

**Methods:**

In our study, we randomly assigned 22 pregnant women with fasting glucose concentrations, 1 hour oral glucose tolerance test (1H-OGTT) and 2 hour oral glucose tolerance test (2H-OGTT), different than 28 normal pregnant women from a sample of 107 pregnant women at the First Affiliated Hospital of Jinan University in China. Lipopolysaccharide (LPS), interleukin 1 alpha (IL-1α), interleukin-6 (IL-6), interleukin-8 (IL-8) and tumor necrosis factor alpha (TNF-α) were measured from blood plasma of pregnant women and umbilical arteries using ultraviolet spectrophotometry. Hematoxylin & Eosin (H&E), Periodic acid-Schiff (PAS) or Masson staining were performed to examine whether diabetes mellitus altered the morphology of placenta. Quantitative PCR (Q-PCR), western blotting and immunofluorescent staining were performed to examine whether diabetes mellitus and autophagy altered the gene expressions of the placental tissue.

**Results:**

We found that women with GDM exhibited increased placental weight and risk of neonatal infection. The concentrations of IL-6 protein and IL-8 protein in GDM were increased in both maternal and umbilical arterial blood. H&E, Masson and PAS staining results showed an increased number of placental villi and glycogen deposition in patients with GDM, but no placental sclerosis was found. Q-PCR results suggested that the expression levels of HIF-1α and the toll like receptor 4 (TLR4)/ myeloid differential protein-88 (MyD88)/ nuclear factor kappa-B (NF-κB) pathway were increased in the GDM placenta. Through Western Blotting, we found that the expression of NF-kappa-B inhibitor alpha (IKBα) and Nuclear factor-κB p65 (NF-κB p65) in GDM placenta was significantly enhanced. We also showed that the key autophagy-related genes, autophagy-related 7 (ATG7) and microtubule-associated protein 1A/1B-light chain 3 (LC3), were increased in GDM compared with normal pregnant women.

**Conclusions:**

Our results suggest that women with GDM exhibit an increased risk of neonatal infection via inflammation and autophagy in the placenta.

## Abstract 2

Supplemental Digital Content is available in the text

## 1 Introduction

According to the eighth edition of the diabetes map released by the International Diabetes Federation in December 2017, and the number of affected newborns has reached 210 million.^[1]^ GDM poses numerous problems for both maternal and fetal outcomes.^[2]^ GDM patients are more likely to suffer from metabolic diseases, such as cardiovascular disease and diabetes, after delivery.^[3]^ For example, pregnant women with GDM are 7 times more likely to develop type 2 diabetes mellitus after delivery than women without GDM.[^4^,^5^] GDM is one of the most common pregnancy complications, and it is generally accompanied by polyhydramnios, premature delivery, stillbirth, fetal malformation, increased cesarean section rate, shoulder dystocia, neonatal infection, hyperbilirubinemia, increased incidence of high blood pressure, myocardial damage, macrosomia, neonatal hypoglycemia, etc.^[6]^

The placenta is a complex fetal organ, as the placenta grows, the risk of insulin resistance becomes greater.^[7]^ Placenta also expresses virtually all known cytokines including TNF-α, interleukin family,^[8]^ which are able to influence the autophagy.^[9]^ GDM is not only a metabolic disease but is also a low-grade inflammatory response.^[10]^ The inflammatory response is characterized by the coordinated activation of various signaling pathways that regulate the expression of pro- and anti-inflammatory mediators in resident tissue cells and leukocytes recruited from the blood.^[11]^ The NF-κB pathway plays a central role in inflammation through its ability to induce the transcription of proinflammatory genes.^[12]^ Both the NF-κB pathway and the autophagy process are involved in several cellular functions, and both processes are necessary for the maintenance of cellular homeostasis. However, the precise molecular mechanisms of the NF-κB pathway and autophagy in placenta are not clear.

The population in the present study was selected from the First Affiliated Hospital of Jinan University in China. We randomly assigned pregnant women to groups; the groups only differed by fasting glucose concentration, 1 hour oral glucose tolerance test (1H-OGTT) and 1 hour oral glucose tolerance test (2H-OGTT). We then compared the risk of neonatal morbidity between women with and without GDM; the outcomes and possible mechanisms induced by the placenta will be discussed.

## 2 Methods

### 2.1 Trial design and participants

**Figure 1:**
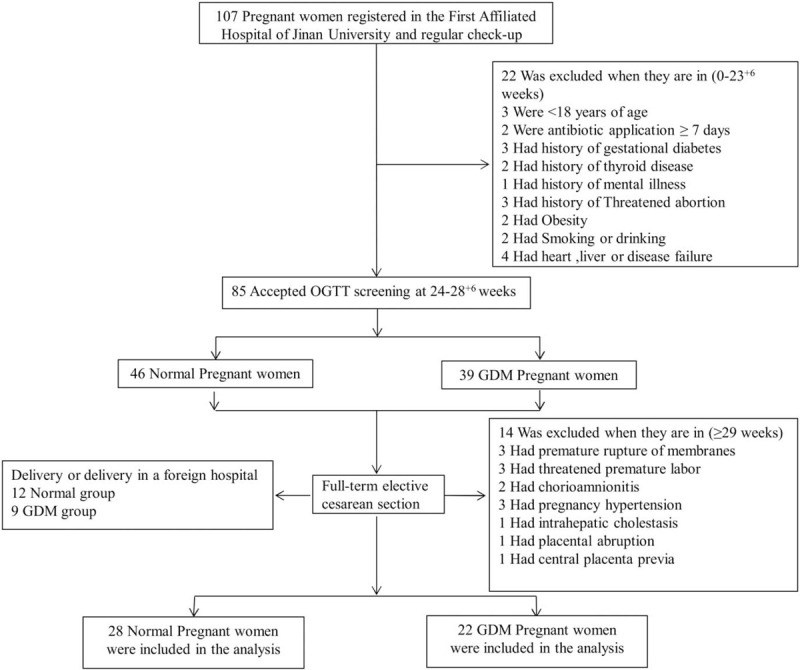
Screening, randomization and analysis of populations.

In this study, we selected 107 pregnant women who were regularly examined at the First Affiliated Hospital of Jinan University from November 2017 to January 2018. In order to prevent vaginal contamination to placenta during childbirth, we chose pregnant woman who planned to deliver via cesarean section. The exclusion criteria were as follows: age <18 years old; a history of antibiotic application longer than 7 days; a history of gestational diabetes; thyroid disease; mental illness; heart, liver or kidney failure; premature birth history; chorioamnionitis; premature rupture of membrane; intrahepatic cholestasis during pregnancy; central type placenta previa; hypertensive disorder complicating pregnancy; placental abruption; and finally, delivery in another hospital. All the women who agreed to participate in the trial provided written informed consent. Finally, 50 pregnant women were registered to our study. This study has been approved by the Ethics Committee of the First Affiliated Hospital of Jinan University. The screening process is shown in Figure 1.

### 2.2 Plasma collection and endotoxin determination

Blood samples were collected from pregnant women and umbilical arteries during childbirth. Plasma was separated from the blood by centrifugation at room temperature and was stored at −80°C. Plasma endotoxin levels were measured using an endotoxin detection kit (Xiamen Horseshoe Crab Reagent Manufactory Co., Ltd, China); IL-1α (CUSBIO, Wuhan, China), IL-6 (CUSBIO, Wuhan, China), IL-8 (CUSBIO, Wuhan, China), TNF-α (CUSBIO, Wuhan, China) were measured using ultraviolet spectrophotometry.

### 2.3 Placental morphological analysis

Morphological analyses were performed to examine whether diabetes mellitus altered the morphology of the placenta. Placentas were fixed in 4% paraformaldehyde (PFA), dehydrated, embedded in paraffin wax and serially sectioned at 4 μm. Sections for histology were dewaxed in xylene, rehydrated and stained with Hematoxylin & Eosin (H&E), Periodic acid-Schiff (PAS) or Masson dyes. The sections were photographed using a fluorescence microscope (Olympus IX50) linked to NIS-Elements F3.2 software. Select a well-stained slice for each specimen, each slice

Randomly select 4 fields of view at 200× field of view, according to stereology method,^[13]^ using the grid test system to count the number of villi. Three different positive fields (200×) were selected for each slice, and Masson staining was positive for blue collagen deposition. Analysis with IPP 6.0 image analysis software, grayscale transformation, blue positive surface and background. Separate, perform quantitative analysis of collagen, calculate the ratio of collagen deposition area of placenta tissue to total area of placental tissue in the field of view, and take the average.

### 2.4 Immunofluorescent staining

Immunofluorescent staining was performed on paraffin vertical sections using antibodies against ATG7 and LC3II. Briefly, the placental vertical sections were dewaxed in xylene, rehydrated and heated in a microwave with citrate buffer (pH = 6.0) for antigen retrieval before exposure to the primary antibodies. Then, sections were immersed in 3% hydrogen peroxide for 10 minutes to block endogenous peroxidase. Unspecific immunoreactions were blocked using 5% inactivated goat serum in PBS for 30 minutes at room temperature. The sections were incubated with ATG7 (1:500, Sigma, USA) or LC3II (1:200, Cell Signaling Technology, USA) antibodies overnight at 4°C. Sections were washed extensively and incubated with the corresponding Alexa Fluor 555 or 488 secondary antibody (1:1000; Invitrogen, Waltham, MA, USA) and DAPI (5 μg/ml, Invitrogen) at room temperature for 2 hours in a dark box.

### 2.5 RNA isolation and quantitative PCR

Total RNA was isolated from the placental tissue using a Trizol kit (Invitrogen, USA) according to the manufacturer's instructions. First-strand cDNA synthesis and the SYBR Green Q-PCR assay were performed using the PrimeScript RT reagent kit (Takara, Japan); and specific primers are described in Supplementary Figure 1. Reverse transcription and amplification reactions were performed in Bio-Rad S1000 (Bio-Rad, USA) and ABI 7000 thermal cyclers, respectively. The expression of genes was normalized to β-actin (a human gene).

### 2.6 Western Blotting

Western blotting was executed with standardized steps, using specific recognition of polyclonal antibodies such as β-actin, IKBα, NF-κB p65, ATG7, LC3-I, LC3-II. Extraction proteins in placental tissue and immunoblotting techniques used were the same as described in previous experiments. Protein from the placenta was isolated from tissue homogenates with lysis buffer (RIPA, Sigma, MO, USA) supplemented with protease and phosphatase inhibitors. Measurement of extracted protein concentration using BCA assay. Separation of extracted proteins by 10% SDS-PAGE and transferred onto a polyvinylidene difluoride (PVDF) membrane (Millipore, MA, USA). After blocking the membrane with 5% non-fat milk and then incubated with β-actin (Abcam, USA), IKBα (Cell Signaling Technology, USA), NF-κB p65 (Cell Signaling Technology, USA), ATG7 (Sigma, USA), LC3-I/LC3-II (Cell Signaling Technology, USA), overnight at 4°C. The membranes were incubated with the secondary antibody (1:3000, EarthOx, 7074S, Millbrae, USA). The blots were developed with the Super Signal West Femto Chemiluminescent Substrate (Thermo Fisher, Rockford, USA), Gel Doc XR+ System (BIO-RAD, CA, USA). Analyze strip intensity using Quantity One software (BIO-RAD, CA, USA) according to the manufacturers instructions. The Western blotting results were representative of 3 independent experiments.

### 2.7 Ethical review

Our research was reviewed and approved by Medical research ethics review committee of the First Affiliated Hospital of Jinan University. The study also obtained informed consent from enrolled patients.

### 2.8 Data analysis

The percentage of Masson-stained areas were quantified and analyzed as follows: The areas occupied by the Masson stain were quantified using an IPP 6.0 image analysis program. The percentage of the Masson-stained area was expressed as the percentage of area occupied by the Masson stain over the entire area under the microscopic field. Statistical analyses for all experimental data generated were performed using the SPSS 13.0 statistical package program for Windows. The data were presented as the means ± SD. Statistical significance was determined using the independent samples *t*-test and the Chi-Squared test. ^∗^ *P* < .05, ^∗∗^ *P* < .01 and ^∗∗∗^ *P* < .001 indicate significant differences between normal and diabetes mellitus groups.

## 3 Results

### 3.1 Clinical characteristics of the study population

By screening a total of 22 cases of GDM and 28 normal cases of pregnancy ultimately entered the study. The screening process is shown in Figure 1. Analysis of the clinical characteristics of the 2 groups during their first trimester found no significant differences in age, weight, height or BMI (body mass index) between the 2 groups (Table 1). We then analyzed the routine blood examinations during the second and the third trimesters in these 2 groups. We found no significant differences in weight, BMI or routine blood examinations between the 2 groups, except for their fasting glucose concentration as determined by 1H-OGTT and 2H-OGTT (Tables 2 and 3).

**Table 1:**
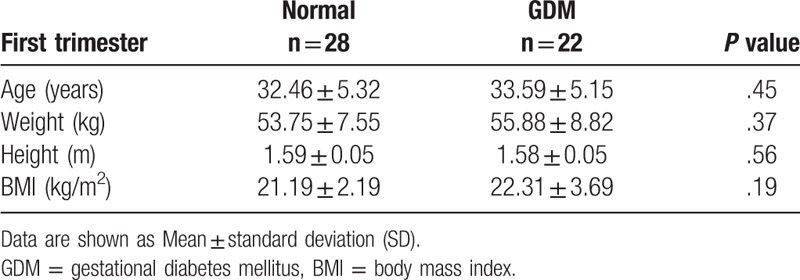
Clinical characteristics of pregnant women with first trimester.

**Table 2:**
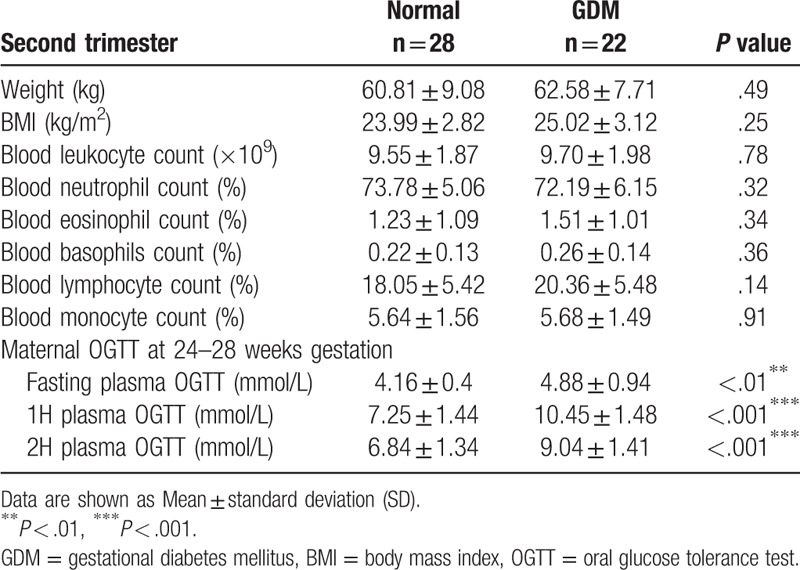
Clinical characteristics of pregnant women with second trimester.

**Table 3:**
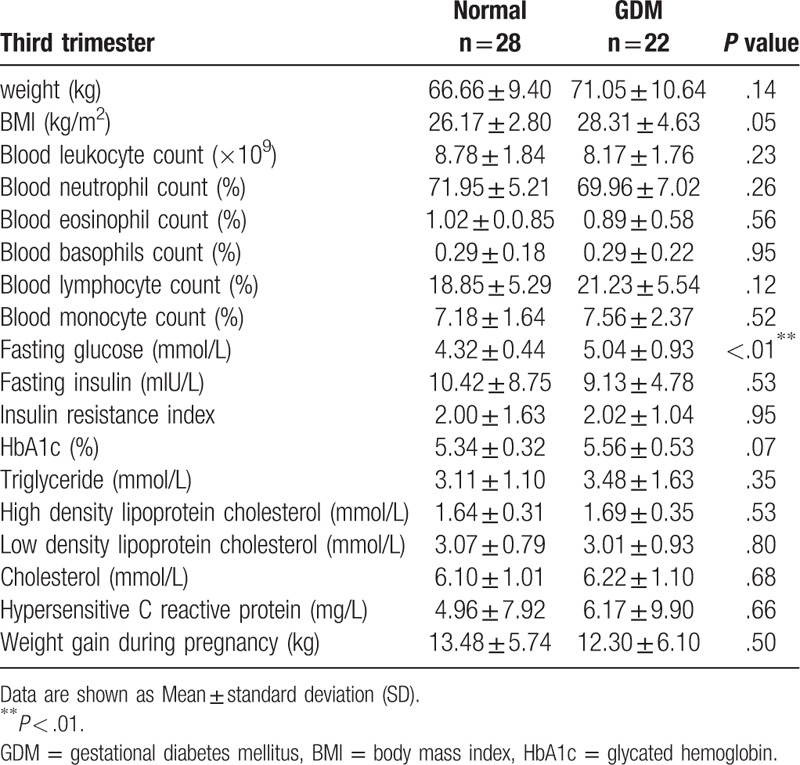
Clinical characteristics of pregnant women with third trimester.

We followed up the pregnancy outcomes and found that only placental weight was significantly higher in the GDM group than the normal group; there were no significant differences in gestational age, birth weight, birth height, birth weight standard deviation score or birth random blood sugar between the 2 groups (Table 4). We also investigated whether the risk of neonatal morbidity was higher in GDM outcomes compared with the normal group. We found that the rate of hyperbilirubinemia and neonatal infection were significantly higher in GDM outcomes than the normal group. There were no significant differences in patent ductus arteriosus, neonatal anemia, low birth weight newborns, hypokalemia or macrosomia. However, during the follow-up, we observed a higher neonatal infection rate in the GDM group (Table 5).

**Table 4:**
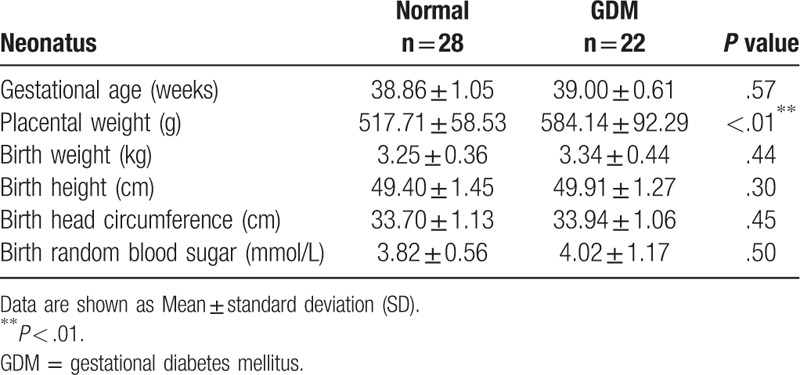
Pregnancy outcomes, according to study group.

**Table 5:**
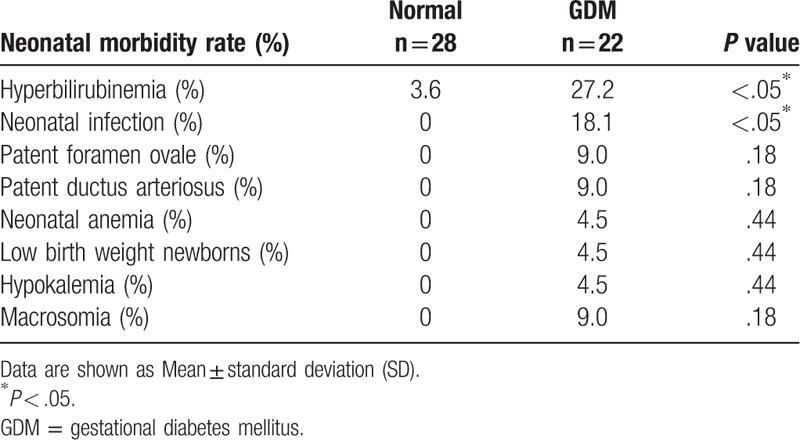
Neonatal morbidity, according to study group.

**Figure 2:**
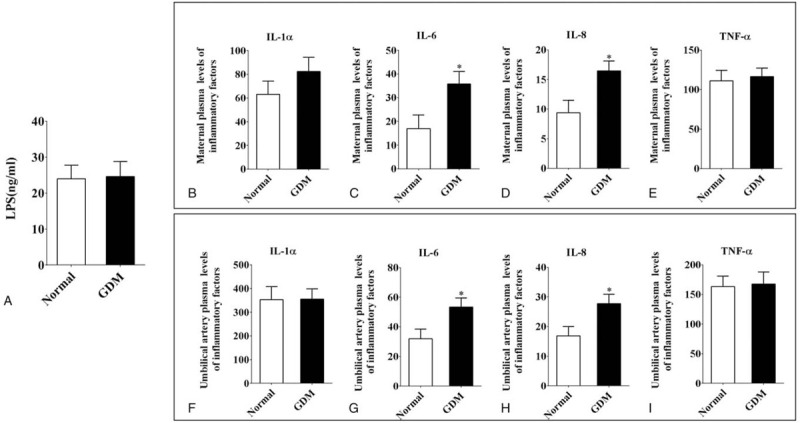
The comparison of the inflammatory factors in maternal and umbilical artery blood plasma. A: Mean plasma LPS values (ng/ml) from normal and GDM women. B-E: The ELISA data showing the expression of the inflammatory factors: IL-1α (B), IL-6 (C), IL-8 (D) and TNF-α (E) in maternal blood plasma. F-I: The ELISA data showing the expression of the inflammatory factors: IL-1α (F), IL-6 (G), IL-8 (H) and TNF-α (I) in umbilical artery blood plasma. ^∗^ *P* < .05.

We analyzed the mechanisms of neonatal infection. We detected LPS, which is a well-known inflammation inducer, in maternal plasma. The LPS level was increased in the GDM group, but no significant differences were observed between the normal and GDM groups (Fig. 2A). Next, we analyzed the inflammatory factors in maternal and umbilical artery plasma using ELISA. The results showed that there are no significant changes in IL-1α in maternal and umbilical cord plasma between the normal and GDM groups (Fig. 2B and F). However, IL-6 and IL-8 in both maternal and umbilical artery plasma were significantly increased in the GDM group compared with normal group (Fig. 2C-D and G-H). However, there were no significant changes in TNF-α in maternal and umbilical artery plasma (Fig. 2E and I).

### 3.2 The placenta villi are increased in GDM compared with normal women

**Figure 3:**
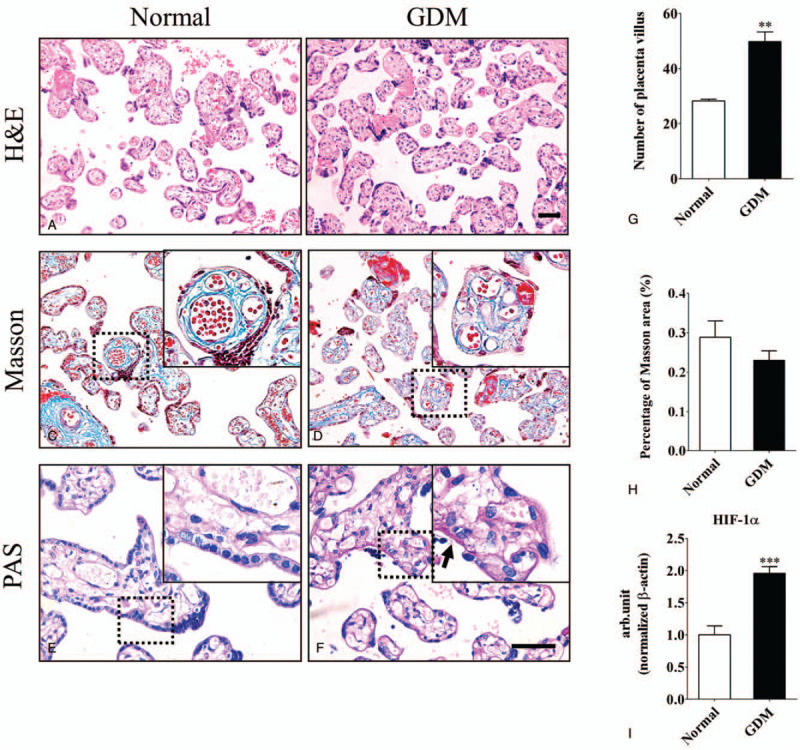
Comparison of the placental morphology in normal and GDM women. A-F: Representative micrographs of normal and GDM placental sections stained with H&E (A-B), Masson (C-D) and PAS (E-F). The top right corner panels in C-F are higher magnification images of the regions highlighted by the dotted squares, respectively. G-H: Bar charts comparing the number of placental villi (G) and percentage of Masson area (H) in normal and GDM women. I: The quantitative PCR data showing the expression levels of HIF-1α in normal and GDM placentas. ^∗∗^ *P* < .01, ^∗∗∗^ *P* < .001. Scale bar = 50 μm in A-B and 50 μm in C-F.

The placenta not only separates the maternal and fetal circulations but it can also produces molecules, such as IL-6 and IL-8, that independently affect mother and fetus.[^8^,^14^,^15^,^16^,^17^,^18^] We detected the placental morphology of normal and GDM groups. Calculated by counting the number of villi in each field of view. We found the number of placenta villi increased in GDM compared with normal group (normal = 28.25 ± 0.70, GDM = 49.79 ± 3.60, n = 10 in each group. *P* < .01. Fig. 3A, B and G). There were no significant differences in the Masson positive areas between the 2 groups (Fig. 3C, D and H). PAS staining revealed heavily stained regions in the GDM groups (black arrow in Fig. 3F) compared with normal group (Fig. 3E). This staining indicates that glycogen synthesis increased in the GDM group. Moreover, the quantitative PCR data revealed that the mRNA level of HIF-1-α in GDM placentas is increased compared with normal group (normal = 1.0 ± 0.10, GDM = 1.96 ± 0.10, n = 3 in each group. *P* < .001. Fig. 3I).

### 3.3 The TLR4/MyD88/NF-κB pathway is affected in the GDM placenta

**Figure 4:**
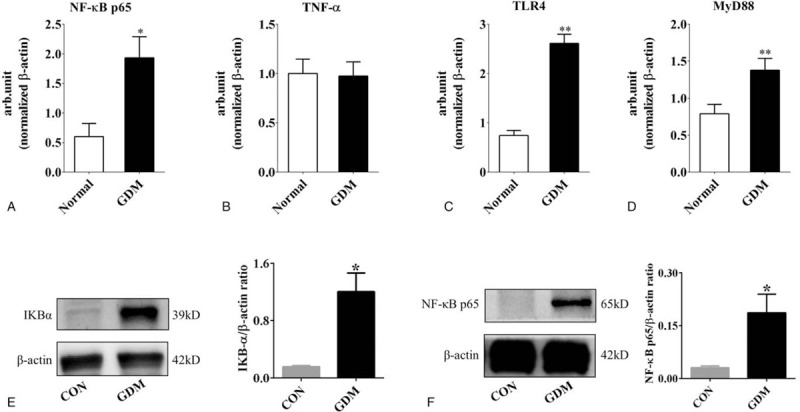
Analysis of inflammatory factors in normal and GDM placentas. A-D: The quantitative PCR data show the gene expression levels of NF-κB p65 (A), TNF-α (B), TLR4 (C) and MyD88 (D) in normal and GDM placentas. E-F: Western blotting data show the gene expression levels of IKBα (E) and NF-κB p65 (F). ^∗^ *P* < .05, ^∗∗^ *P* < .01.

NF-κB signaling plays an important role in the synthesis of inflammatory cytokines and can induce premature birth during an infection.^[19]^ The Q-PCR results showed that NF-κB p65 mRNA was significantly increased in GDM placentas compared with the normal group (normal = 0.60 ± 0.20, GDM = 1.93 ± 0.40, n = 3 in each group. *P* < .05. Fig. 4A). There were no significant differences in TNF-α between the 2 groups (normal = 1.0 ± 0.10, GDM = 0.97 ± 0.10, n = 3 in each group. *P* < .05. Fig. 4B). TLR4 and the downstream MyD88 always lead to activation of NF-κB and the transcription of many genes involved in the inflammatory response.^[20]^ The expression levels of TLR4 mRNA and MyD88 mRNA were significantly increased in GDM placentas compared with the normal group (TLR4: normal = 0.74 ± 0.10, GDM = 2.62 ± 0.20, *P* < .01; MyD88: normal = 0.79 ± 0.10, GDM = 1.38 ± 0.20; n = 3 in each group. *P* < .05. Fig. 4C, D). By western blotting, we discover that the protein expression of IKBα and p65 is significantly enhanced as shown in (Fig. 4E and F).

### 3.4 Autophagy is increased in GDM compared with normal women

**Figure 5:**
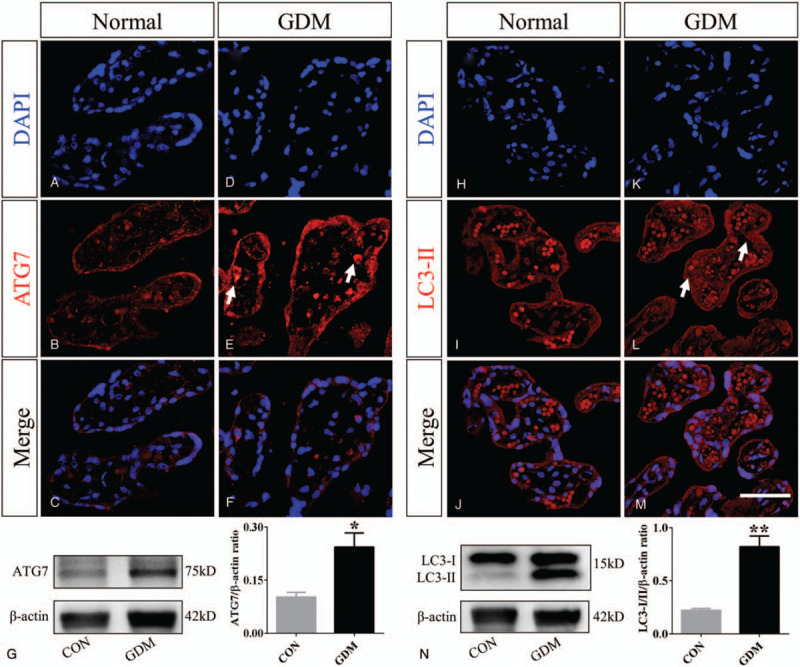
Comparisons of autophagy-related genes in placental villi of normal and GDM groups. A-F: Representative transverse sections of DAPI staining (A and D), ATG7 staining (B and E), and merged images (C and F) that were taken in normal and GDM placentas. G: Western blotting data show the protein expression level of ATG7. H-M: The representative transverse sections of DAPI staining (H and K), LC3II staining (I and L), and merged images (J and M) that were taken in normal and GDM placentas. The white arrows in E and L indicate highly expressing ATG7 and LC3II cells. N: Western blotting data show the protein expression levels of LC3I/II. ^∗^ *P* < .05, ^∗∗^ *P* < .01. Scale bar = 50 μm in A-F and H-M.

We detected the ATG7 and LC3I-II expression levels in normal and GDM placentas (n = 3 in each group. Fig. 5A–F and 5H–M). We observed that ATG7 and LC3I-II were highly expressed in GDM placental villi (arrows in Fig. 5 E and L). Similarly, we also see this phenomenon at the level of protein expression by western blotting (Fig. 5G and N).

## 4 Discussion

Autophagy as a biological process to maintain cell homeostasis played a pivotal role in the formation of the placenta. Autophagy helps the body maintain normal homeostasis to avoid disease, aging, and dysplasia.[^21^,^22^] Autophagy also reflects intracellular metabolic levels such as glucose metabolism.^[23]^ When the intracellular regulation in the placenta is unbalanced, there is an increase in autophagy in the villus trophoblast, such as eclampsia and intrauterine growth restriction.[^24^,^25^] But the development of autophagy in the GDM placenta is still unclear. We speculate that high glucose may increase inflammatory response and autophagy in GDM. The only differences between groups were fasting glucose concentration as determined by 1H-OGTT and 2H-OGTT, implying that the placenta of GDM is in a high sugar environment implying that the placenta of GDM is in a high sugar environment. According to the newborn pediatrician, the newborn after birth presents abnormal symptoms such as not crying, not rising body temperature, not moving, or jaundice further increased and the others,[^26^,^27^] laboratory tests were carried out on the newborns. Some of them were diagnosed with neonatal infections. This is also consistent with the research results of Ana et al.^[28]^ Since the placenta is a vehicle for maternal and infant inflammation.^[29]^ Through statistical analysis we found that the risk of neonatal infection and placenta weight were higher in the GDM than the control group, which suggests that the placenta plays an important role in neonatal infection in women with GDM.

Although the underlying mechanisms are still under investigation, increasing evidence suggests that the systemic and local production/action of inflammation in the placenta may be particularly critical in changing the placental structure.[^30^,^31^] By following up within a week of birth to determine the incidence of neonatal findings, we found that the risk of neonatal infection was increased in GDM fetuses. This increase may be related to the increased expression of IL-6 protein and IL-8 protein in GDM umbilical artery blood (fetus blood, Fig. 2). Through the exchange of substances in the placenta, GDM neonates are experiencing a low-grade inflammatory response in the uterus. IL-8 cannot cross the placenta.^[32]^ But the IL-8 levels in both maternal and umbilical cord plasma were significantly increased in the GDM group, which suggests different placental secretion in GDM women.

We then analyzed placental villi in GDM women and found that the number of villi increased. The basal layer of the GDM placenta maternal decidua was more susceptible to glycogen deposition, as observed using PAS staining (Fig. 3). These findings were similar to previous research.^[33]^ There was no significant change in the Masson positive areas between the 2 groups, suggesting GDM did not induce placental sclerosis in our research. Bensellam et al suggested that a high-glucose environment activated HIF-1α because of the relatively low-oxygen microenvironment in the placenta.^[34]^ HIF-1α binds to a specific promoter of TLR4 and upregulates the expression of TLR4 under hypoxic conditions.[^35^,^36^,^37^] Activation of toll-like receptor 4 (TLR4) can cause the expression of NF-κB in cells and can cause the release of inflammatory factors, such as interleukin-6 (IL-6) and interleukin-8 (IL-8).[^15^,^38^,^39^] TLR4 is primarily expressed in vascular endothelial cells, trophoblast cells and the placenta, and the expression of TLR4 in trophoblasts is higher than it is in vascular endothelial cells. TLR4 in trophoblasts plays a key role in the development of inflammatory metabolism in the placenta.^[21]^ We also detected the TLR4/MyD88/NF-κB pathway in the placentas because TLR4/MyD88/NF-κB with insulin resistance in placentas of GDM act as a key regulatory factor.[^40^,^41^]

The NF-κB system consists of the NF-κB family and its inhibitor IKB family. The former is composed of members of the Rel protein family and exists as homologous or heterodimeric forms. Activation of NF-κB must be dissociated and degraded from the NF-κB complex by IKB, exposing the nuclear localization sequence of NF-κB, then combined with a specific KB sequence such as p65.^[42]^ Through research we found significant activation of IKBα and NF-κB p65 in the placenta of the GDM group. Furthermore, the activity of NF-κB downstream factors can enhance the transcription of HIF-1α.[^43^,^44^,^45^,^46^] We found that this pathway was highly activated in the GDM placenta compared with the normal group (Fig. 4).

TLR4/MyD88/NF-κB signaling-induced inflammatory cytokines have also been linked to autophagy in many kinds of cells and organs including the placenta.[^47^,^48^,^49^] Shi-Fang Z et al demonstrated that ATG7 has proangiogenic activity in brain angiogenesis which is mediated by IL-6 production in a NF-κB-dependent manner.^[13]^ The number of villi in the placental tissue of the GDM group increased, and the increase in vascular density was observed in our research. Furthermore, ATG7 plays an important role in TLR-mediated IL-8 production in intestinal epithelial cells.^[50]^ We found that the key autophagy-related genes, ATG7 and LC3, were increased in women with GDM compared with normal women (Fig. 5). In our previous study, we found that autophagy is involved in diabetes mellitus-induced defects in the development of mouse placenta.^[51]^ Studies have shown that NF-κB is not only a pro-inflammatory factor, but also regulates the process of autophagy. IKK-NF-κB axis is a context-dependent regulator of autophagy^[52]^ ROS may also promote autophagy, and the imbalance of cell autophagy might also contribute to the observed placental phenotypes directly or indirectly.[^51^,^53^,^54^] So there may be a closed loop in the GDM placenta, high glucose stimulates TLR4s pathway, and there is an imbalance between the internal and external environment of placental villus trophoblast cells. In order to suppress this imbalance, autophagy is up-regulated.

## 5 Limitation

First of all, Due to the strict screening conditions, the number of cases included in our experiment is not large enough. Secondly, the classification of neonatal infections was missing in this study. Moreover, the neonates involved in this experiment were only followed up for a short time. We will focus on the research of different types of neonatal infection effects on neonatal outcome and more precise molecular biological mechanisms in GDM placentas require further investigation in the future.

## 6 Conclusions

Overall, our study showed that GDM women exhibited increased risk of neonatal infection because of an abnormal placenta. The high-glucose environment activated HIF-1α and the TLR4/MyD88/NF-κB pathway in GDM placentas and induced the secretion of the IL-6 and IL-8. Inflammation caused by GDM breaks the homeostasis in the placenta, and autophagy balance is also destroyed by maintaining cell self-renewal and maintaining homeostasis. Autophagy was increased in GDM compared with normal women. Furthermore, GDM neonates experienced a low-grade inflammatory response in the uterus and had increased risk of neonatal infection after birth.

## Acknowledgments

Thanks to all the women and newborns who participated in this study. I would like to thank the obstetricians and neonatologists of the first affiliated hospital of Jinan University for their support and assistance in this study.

## Author contributions

**Conceptualization:** Deng-lu Long, Di Qiu.

**Funding acquisition:** Xuesong Yang, Xin Cheng, Ruiman Li, Guang Wang.

**Methodology:** Jingyun Wang.

**Resources:** Jia Liu.

**Writing – original draft:** Yixiao Li.

**Writing – review & editing:** Ruiman Li, Guang Wang.

## Supplementary Material

Supplemental Digital Content
